# SOD2 Mediates Amifostine-Induced Protection against Glutamate in PC12 Cells

**DOI:** 10.1155/2016/4202437

**Published:** 2015-12-07

**Authors:** Ji Jia, Lei Zhang, Xiaolei Shi, Mingchun Wu, Xiang Zhou, Xiaonan Liu, Tingting Huo

**Affiliations:** ^1^Department of Anesthesiology, Xijing Hospital, Fourth Military Medical University, Xi'an 710032, China; ^2^Department of Anesthesiology, Xi'an No. 4 Hospital, Xi'an 710032, China; ^3^Department of General Surgery, Fuzhou General Hospital of Nanjing Military Command, Fuzhou 350025, China; ^4^Department of Anesthesiology, Wuhan General Hospital of Guangzhou Military Command, Wuhan 430070, China; ^5^Department of Gastrointestinal Surgery, Xijing Hospital of Digestive Diseases, Fourth Military Medical University, Xi'an 710032, China

## Abstract

*Background*. Cytoprotectant amifostine attenuates radiation-induced oxidative injury by increasing intracellular manganese superoxide dismutase (SOD2) in peripheral tissue. However, whether amifostine could protect neuronal cells against oxidative injury has not been reported. The purpose of this study is to explore the protection of amifostine in PC12 cells. *Methods*. PC12 cells exposed to glutamate were used to mimic neuronal oxidative injury. SOD assay kit was taken to evaluate intracellular Cu/Zn SOD (SOD1) and SOD2 activities; western blot analysis and immunofluorescence staining were performed to investigate SOD2 protein expression; MTT, lactate dehydrogenase (LDH), release and cell morphology were used to evaluate cell injury degree, and apoptotic rate and cleaved caspase-3 expression were taken to assess apoptosis; mitochondrial superoxide production, intracellular reactive oxygen species (ROS), and glutathione (GSH) and catalase (CAT) levels were evaluated by reagent kits. *Results*. Amifostine increased SOD2 activity and expression, decreased cell injury and apoptosis, reduced mitochondrial superoxide production and intracellular ROS generation, and restored intracellular GSH and CAT levels in PC12 cells exposed to glutamate. SOD2-siRNA, however, significantly reversed the amifostine-induced cytoprotective and antioxidative actions. *Conclusion*. SOD2 mediates amifostine-induced protection in PC12 cells exposed to glutamate.

## 1. Introduction

Oxidative stress induces a variety of neurological disorders, including brain ischemia, brain trauma, and neurodegenerative conditions [1–3]; therefore, inhibiting oxidative stress-induced neuronal injury is regarded as an effective therapy for these diseases. Cells prevent oxidative injury by utilizing several antioxidant systems, which resist oxidative adversity by eliminating intracellular reactive oxygen species (ROS) [4]. Superoxide dismutase (SOD) is a vital antioxidative system, including SOD1, SOD2, and SOD3. Utilizing Cu or Zn as its prosthetic group, SOD1 (Zn/Cu SOD) is localized in cytosolic compartments, while SOD3 (Ec-SOD) is found in extracellular spaces. On the contrary, SOD2 uses Mn as its active site, which is localized strictly in the mitochondria [5, 6]. And overexpression of SOD2 can protect neurons from lethal consequences of oxidative damage both* in vitro* and* in vivo* [7, 8]. Its primary function is to facilitate the dismutation of two molecules of superoxide anion into water and hydrogen peroxide [9].

Amifostine is a cytoprotectant, commonly used to alleviate the side effects of radiotherapy and chemotherapy in treating malignant tumors [10, 11]. Grdina et al. reported that amifostine protects normal tissue against radiation-induced injury by increasing intracellular SOD2 activity [12]. Moreover, Murley et al. found that WR1065, the active free thiol form of amifostine, induces antioxidative ability against radiation via SOD2* in vitro* [13]. However, whether amifostine could induce protection against oxidative injury in central nervous system (CNS) has not been investigated.

Glutamate is an excitatory neurotransmitter, which causes neurotrophic effect in physiological concentrations [14]. However, in pathological conditions, including brain ischemic injury, traumatic brain injury, and Parkinson's disease, the concentration of glutamate in brain may increase and result in oxidative injury to neuronal cells ultimately [15–17]. High level of glutamate in brain consumes neuronal antioxidase and increases reactive oxygen species (ROS) accumulation, leading to an imbalance of intracellular antioxidants and oxidants [18, 19]. Thus, neuronal cells exposed to high concentration of glutamate are used widely in investigating oxidative stress injury of neurological conditions [20]. PC12 cell is a rat pheochromocytoma cell-line, used commonly in the study of neurons [21], especially dopaminergic neurons* in vitro*. And PC12 cells exposed to glutamate have been taken universally to investigate oxidative stress injury of neurological diseases [22, 23].

In this study, we used PC12 cells exposed to glutamate to mimic oxidative stress injury of neurons and hypothesized that amifostine could exert protection against glutamate in PC12 cells, and the protection is mediated by SOD2.

## 2. Materials and Methods

### 2.1. Materials

The PC12 cell-line was a gift from the Shanghai Institute of Cell Biology, Chinese Academy of Sciences (Shanghai, China). Dulbecco's Modified Eagle's Medium (DMEM), fetal bovine serum (FBS), 3-(4,5-dimethyl-2-thiazolyl)-2,5-diphenyl-2-tetrazolium bromide (MTT), and nerve growth factor (NGF) were purchased from Sigma-Aldrich (St. Louis, MO, USA). The primary anti-SOD2 and anti-cleaved caspase-3 antibodies were purchased from Santa Cruz (USA). MitoTracker and MitoSOX staining kits were purchased from Invitrogen Molecular Probes (San Diego, CA). The 4′,6-diamidino-2-phenylindole (DAPI) staining solution and ROS reagent kit were purchased from Beyotime Technology (Nantong, China). The SOD activity assay kit was purchased from Trevigen (Gaithersburg, USA). The LDH, GSH, and CAT reagent kits were purchased from Nanjing Jiancheng Bioengineering Institute (Nanjing, China).

### 2.2. Cell Culture

PC12 cells were cultured at 37°C in an atmosphere of 5% CO_2_ and 95% air in DMEM medium. The DMEM medium was supplemented with 10% (v/v) of heat-inactivated horse serum (Sigma, St. Louis, MO, USA), 5% (v/v) FBS, 100 U/mL penicillin, and 100 *μ*g/mL streptomycin. To differentiate the PC12 cells into neuron-like cells, DMEM containing 1% (v/v) FBS, 100 U/mL penicillin, and 100 *μ*g/mL streptomycin was supplemented with 100 ng/mL of NGF for 3 days.

### 2.3. Cell Viability Assay

PC12 cells were seeded at a density of 1 × 10^4^ cells/well in a 96-well plate. After treatments, MTT solution (20 *μ*L, 5 mg/mL) was added to each well, and after 4 h incubation at 37°C, the supernatant of each well was removed. Dimethyl sulfoxide (DMSO) of 150 *μ*L was added to each well to solubilize the formazan product. The plate was then shaken for 10 min until all the formazan had completely dissolved. The absorbance was then evaluated at a wavelength of 490 nm by using a spectrophotometer (TECAN, CH).

### 2.4. SOD1 and SOD2 Activities Assay

PC12 cells were seeded into a 6-well plate at a density of 1 × 10^5^ cells/well. After being treated with different concentrations of amifostine for 24 h, 500 *μ*L of lysis solution was added to the well. After 5 min, the resulting suspension was centrifuged at 14,000 g for 5 min at 4°C and then transferred to a clean 2-mL tube. Protein concentration was determined by using the Bradford assay and adjusted to 5 *μ*g/*μ*L with lysis buffer. Total SOD activity was determined at room temperature using a colorimetric assay based on the ability of SOD to form H_2_O_2_ from superoxide radicals generated by an exogenous reaction involving xanthine and xanthine oxidase that can convert nitroblue tetrazolium (NBT) to NBT-diformazan. The extent of reduction in the appearance of NBT-diformazan is a total SOD activity. Activity was measured using 50–500 *μ*g of total cellular protein in the presence of 5 mM sodium cyanide (NaCN) (Sigma-Aldrich, USA) to inhibit Cu/ZnSOD (SOD1) activity. Absorbance changes were recorded for 5 min, and the rate of increase in absorbance per minute was calculated for each experimental sample and a negative control, which contained all of the reaction components except for cell lysate. The percentage inhibition was calculated and plotted as a function of protein concentration for each group. The highest maximum percentage inhibition for each group curve to be compared was evaluated and used to calculate the amount of protein that inhibited NBT reduction by 50% of this maximum value. Total SOD (reactions not containing NaCN) and SOD2 activities (reactions containing 5 mM NaCN) in U/mg protein were then calculated. SOD1 activities were determined by subtracting the SOD2 activity in each group from the total SOD activity.

### 2.5. SOD2-siRNA Interfering

SOD2-siRNA and scrambled- (SC-) siRNA were purchased from QIAGEN (Germany). The sentence and anti-sentence were as follows: 5′-GGCUUACUAUUAAACAUUATT-3′ and 5′-UAAUGUUUAAUAGUAAGCCTA-3′. Briefly, SOD2 and SC-siRNA oligomers were diluted in DMEM medium without FBS and antibiotics and incubated for 5 min at room temperature. The SC-siRNA served as the negative control. The oligomers were then combined with diluted Lipofectamine 2000, mixed gently, and incubated for 20 min to allow complex formation. During this incubation, the cell culture medium was removed from the plate and the cells were washed twice with phosphate buffered saline (PBS) at 37°C to remove the traces of FBS. The siRNA-Lipofectamine complexes were added to the plate. The cells were incubated for 24 h at 37°C. Then, the cells were washed with PBS and incubated in the medium containing different drugs for 24 h at 37°C.

### 2.6. Immunocytochemistry

PC12 cells were seeded into a confocal microscopy special dish at a density of 2 × 10^4^ cells/dish. After treatments, the cell culture medium was replaced by the medium containing 200 nM MitoTracker probe (red, excitation = 579 nm, emission = 599 nm). After an incubation of 30 min at 37°C, the cells were washed three times with PBS in the dark and fixed with 4% paraformaldehyde solution for 1 h at room temperature. Then, 2 mL BSA solution (50 mg/mL in PBS) was added to the dish to block the fixed cells. Then, the cells were incubated with SOD2 primary antibody (1 : 100) at 4°C for 24 h. Then, Cy3-labeled secondary antibody (1 : 200, green, excitation = 490 nm, emission = 520 nm) was added to the dish. After 2-h incubation at room temperature in the dark, 100 *μ*L DAPI solution was added to the dish. Five min later, the dish was washed three times with PBS. Then, the SOD2 protein expression and mitochondria staining in PC12 cells were observed by a confocal microscope.

### 2.7. Western Blot Analysis

After the treatments, PC12 cells were lysed with modified RIPA-buffer containing a protease inhibitor-cocktail and 100 *μ*M phenylmethanesulfonyl fluoride on ice for 30 min. The total protein content was qualified by a bicinchoninic acid kit. Total protein lysates were subjected to 12% sodium dodecyl SDS-PAGE and transferred onto polyvinylidene difluoride membranes. Membranes were incubated with rabbit anti-mouse primary antibody (SOD2, 1 : 500; cleaved caspase-3, 1 : 500) in PBS with 0.1% Tween-20 overnight at 4°C and then incubated for 1 h at room temperature with anti-rabbit IgG. *β*-tubulin served as the control. Expression was visualized by enhanced chemiluminescence. The signal was quantified by densitometry by an immunoblotting detection system (Alpha Innotech, USA).

### 2.8. LDH Release Assay

PC12 cells were seeded at a density of 2 × 10^4^ cells/well into a 24-well plate. After treatments, the supernatants of each well were removed for evaluation of LDH release, which was measured as described previously. Briefly, 100 *μ*L of cell-free supernatant, 250 *μ*L of buffer, and 50 *μ*L of coenzyme were mixed homogeneously. Then, the mixture was incubated for 15 min at 37°C. Then, 250 *μ*L of 2,4-dinitrophenylhydrazine was added to the mixture and incubated for an additional 15 min at 37°C in the dark. Finally, 2.5 mL of NaOH (400 mM) was added to the mixture to stop the reaction. After 3 min, the absorbance of the mixture was evaluated at 440 nm by spectrophotometry. The absorbance of the sample blank, standard, and standard blank was measured simultaneously. LDH activity was calculated according to the following formula: LDH activity (U/L) = [(sample  OD − sample  blank  OD)/(standard  OD − standard  blank  OD)] × 2 × 1000 U/L.

### 2.9. Apoptotic Rate Assay

PC12 cells were seeded into a 6-well plate at a density of 1 × 10^5^ cells/well. After treatments, the PC12 cells were harvested by centrifugation at 1000 rpm for 5 min. After two washes with cold PBS, the cells were resuspended in binding buffer at a density of 1 × 10^6^ cells/mL. Then, 5 *μ*L of fluorescein 5-isothiocyanate [2-(3,6-dihydroxy-9H-xanthen-9-yl)-5-isothiocyanatobenzoic acid] of FITC-conjugated anti-annexin-V staining antibody and 2 *μ*L of propidium iodide (PI) solution were added to 100 *μ*L of the binding buffer. After completely mixing, the cells were incubated for 15 min at room temperature in the dark, and the apoptotic rates were assessed by flow cytometry (BD, USA).

### 2.10. Mitochondrial Superoxide Measurements

MitoSOX Red reagent was used to measure mitochondrial superoxide level. MitoSOX does not react with intracellular ROS and reactive nitrogen species, but it targets the mitochondria selectively after permeating cell membrane and reacting with the mitochondrial superoxide rapidly. After the treatments, PC12 cells were incubated with 5 *μ*M MitoSOX Red for 20 min at 37°C. A confocal microscope (Olympus) was used to take the fluorescence photographs of PC12 cells, including mitochondrial superoxide (red, 510 nm at excitation and 580 nm at emission), nuclei (blue, 340 nm at excitation and 488 nm at emission), and the merged photos. Then, fluorescence intensities of the mitochondrial superoxide photographs were evaluated by using image pro-plus software (IPP 6.0, Media Cybernetics, Silver Spring, MD, USA).

### 2.11. Intracellular ROS Assay

PC12 cells were seeded into confocal microscopy special dishes at a density of 2 × 10^4^ cells/well. After the treatments, the level of intracellular ROS was assessed by a ROS assay kit. This assay was based on the oxidation of nonfluorescent and colorless DCF-DA to the fluorescent 2′,7′-dichlorofluorescein (DCF) by intracellular production of ROS. PC12 cells were incubated with 100 *μ*M DCF-DA (dissolved in DMEM without FBS) for 20 min at 37°C. After being washed three times with PBS, fluorescence photographs of the cells were taken by using a confocal microscope (480 nm at excitation and 535 nm at emission). Then, fluorescence intensities of DCF photos were evaluated by using image pro-plus software.

### 2.12. Intracellular GSH and CAT Measurements

PC12 cells were seeded into 6-well plates at a density of 5 × 10^5^ cells/well. After the treatments, cells were harvested and homogenized in 0.5 mL of 0.1 M phosphate buffer (pH 7.4). The mixture was centrifuged at 3000 rpm for 10 min at 4°C, and supernatants were used for GSH and CAT activity assessments with the corresponding reagent kits by spectrophotometry.

### 2.13. Statistical Analysis

SPSS 11.0 (SPSS Inc., Chicago, IL) was used to conduct statistical analysis. Values were expressed as means ± standard deviation (SD). Results were compared by one-way ANOVA, followed by Tukey's Multiple Comparison Test. *P* < 0.05 was considered statistically significant.

## 3. Results

### 3.1. Amifostine Attenuated Glutamate-Induced Injury to PC12 Cells

**Figure 1:**
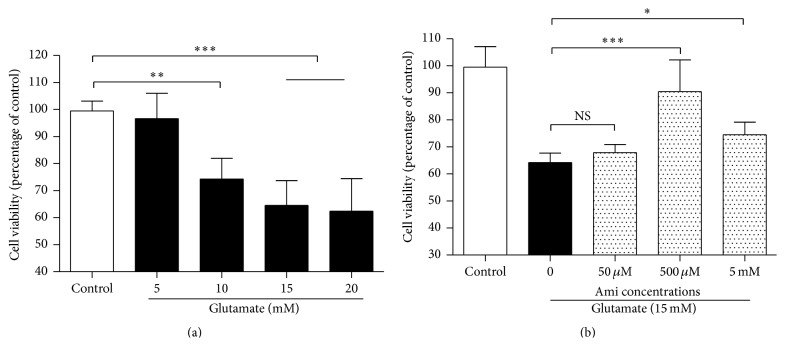
Amifostine protected PC12 cells against glutamate. (a) Glutamate decreased cell viability of PC12 cells in a dose-dependent manner. PC12 cells were exposed to 5 mM–20 mM glutamate for 24 h (*n* = 8). (b) Amifostine restored the cell viability of PC12 cells exposed to glutamate. PC12 cells were exposed to different concentrations of amifostine (Ami) plus 15 mM glutamate for 24 h (*n* = 8). Cell viability was evaluated by MTT assay. Results are means ± SD, ^*∗*^ *P* < 0.05, ^*∗∗*^ *P* < 0.01, ^*∗∗∗*^ *P* < 0.001, and NS: no significance.

To find a moderate cell injury level, PC12 cells were exposed to 5 mM–20 mM glutamate for 24 h (Figure 1(a)), inducing a dose-dependent decrease of cell viability, and 15 mM glutamate was used in the subsequent experiments. Then, the PC12 cells were exposed to 50 *μ*M, 500 *μ*M, and 5 mM amifostine in the presence of 15 mM glutamate (Figure 1(b)); after 24 h incubation, 500 *μ*M and 5 mM amifostine restored the cell viability significantly (*P* < 0.001 and *P* < 0.05, resp.), compared with the cells exposed to glutamate only. Amifostine of 500 *μ*M was taken in the subsequent experiments.

### 3.2. Effect of Amifostine Exposure on SOD Enzymatic Activity

**Figure 2:**
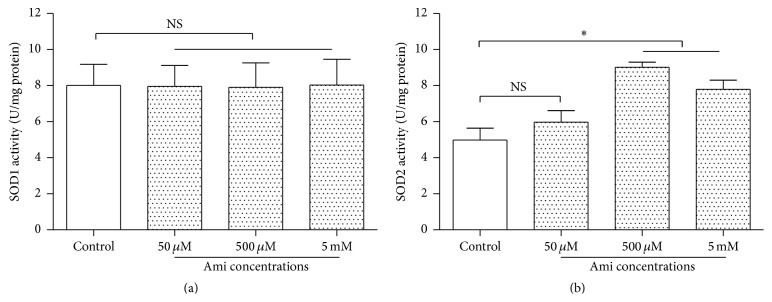
Amifostine exposure increased SOD2 activity in PC12 cells. (a) Amifostine did not induce a significant effect on SOD1 activity (*n* = 6). (b) Amifostine increased SOD2 activity. PC12 cells were exposed to 500 *μ*M amifostine (Ami) for 24 h (*n* = 6). SOD1 and SOD2 activities were evaluated by using a SOD assay kit. Results are means ± SD, ^*∗*^ *P* < 0.05, and NS: no significance.

SOD assay kit was used to determine the cytoplasmic SOD (SOD1) and mitochondrial SOD (SOD2) activities. PC12 cells were exposed to different concentrations of amifostine (50 *μ*M–5 mM) for 24 h, and amifostine exposure did not cause a significant increase of SOD1 activity in PC12 cells (*P* > 0.05), compared with the control (Figure 2(a)). In contrast, an exposure of 500 *μ*M or 5 mM amifostine induced a marked increase of SOD2 activity (Figure 2(b)) in the cells (*P* < 0.05), indicating amifostine exposure increases SOD2 activity, not SOD1, in PC12 cells.

### 3.3. SOD2-siRNA Reversed Amifostine-Induced Upregulation of SOD2 Protein Expression

**Figure 3:**
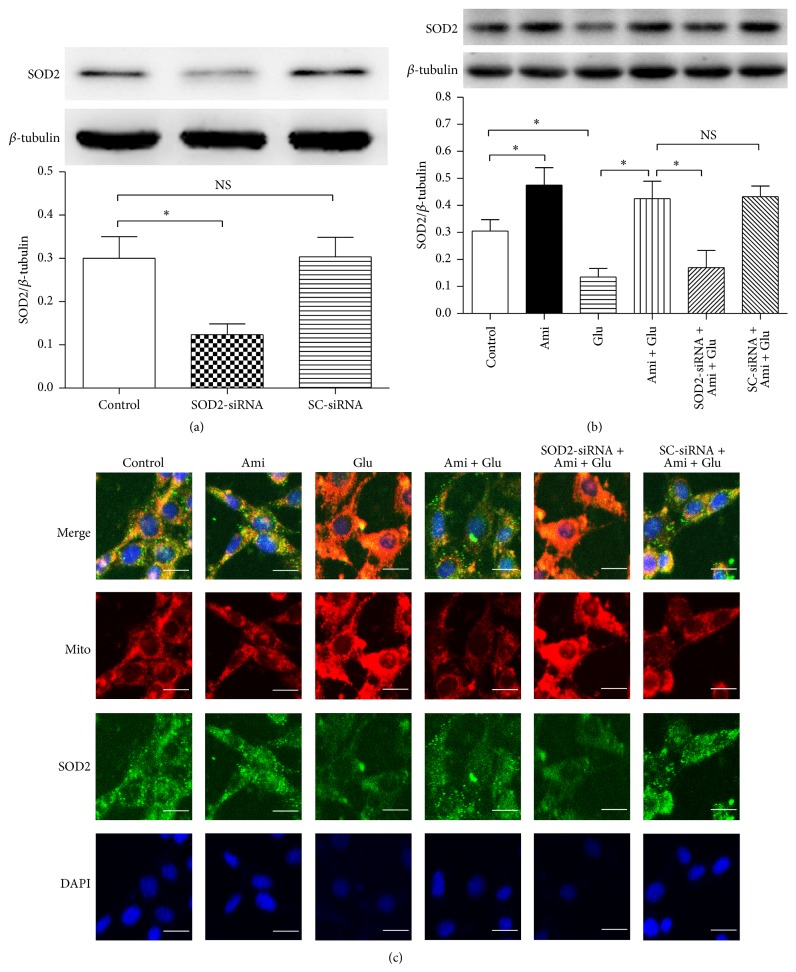
SOD2-siRNA abolished SOD2 protein upregulation induced by amifostine in PC12 cells. The PC12 cells were divided into six groups: control: cells cultured in drug-free medium; Ami: cells exposed to 500 *μ*M amifostine for 24 h; Glu: cells treated with 15 mM glutamate for 24 h; Ami + Glu: cells exposed to 500 *μ*M amifostine plus 15 mM glutamate for 24 h; SOD2-siRNA + Ami + Glu: cells incubated with SOD2-siRNA transfection complexes for 24 h and then exposed to 500 *μ*M amifostine plus 15 mM glutamate for 24 h; SC-siRNA + Ami + Glu: cells incubated with scrambled- (SC-) siRNA transfection complexes for 24 h and then exposed to 500 *μ*M amifostine plus 15 mM glutamate for 24 h. (a) SOD2-siRNA was effective in decreasing SOD2 protein expression. (b)-(c) Amifostine exposure increased SOD2 protein expression, and the effect was reversed by SOD2-siRNA. Western blot analysis and immunofluorescence staining were used to assess SOD2 protein expression. Nuclei were counterstained with DAPI (blue). Results are means ± SD, ^*∗*^ *P* < 0.05, NS: no significance, and bar = 10 *μ*m.

To further investigate the role of SOD2 in amifostine-induced protection in PC12 cells, SOD2-siRNA was used to knock down the expression of SOD2 protein in PC12 cells, and western blot and immunocytochemistry were taken to evaluate the expression level of SOD2. We found that SOD2-siRNA was effective in reducing SOD2 protein expression (Figure 3(a)). In addition, we observed that amifostine obviously upregulated SOD2 protein expression and reversed glutamate-induced reduction of SOD2 expression, and SOD2-siRNA significantly abolished the amifostine-induced effects on SOD2 expression (Figures 3(b) and 3(c)), suggesting amifostine can upregulate the SOD2 expression in PC12 cells.

### 3.4. SOD2 Mediated Amifostine-Induced Protection against Glutamate in PC12 Cells

**Figure 4:**
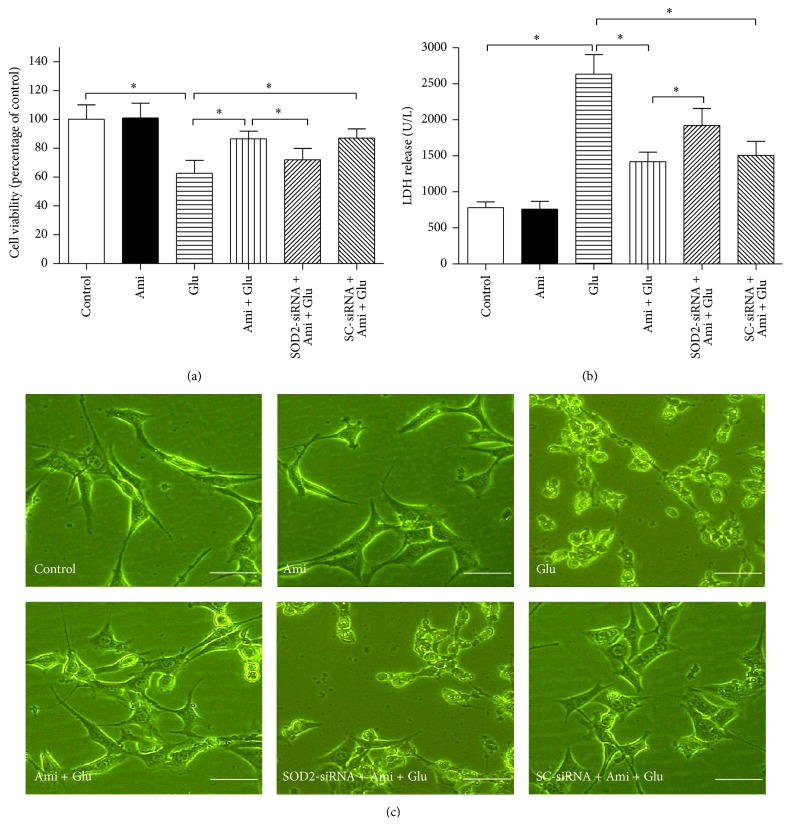
SOD2-siRNA abolished amifostine-induced protection in PC12 cells exposed to glutamate. The PC12 cells were divided into six groups: control: cells cultured in drug-free medium; Ami: cells exposed to 500 *μ*M amifostine for 24 h; Glu: cells treated with 15 mM glutamate for 24 h; Ami + Glu: cells exposed to 500 *μ*M amifostine plus 15 mM glutamate for 24 h; SOD2-siRNA + Ami + Glu: cells incubated with SOD2-siRNA transfection complexes for 24 h and then exposed to 500 *μ*M amifostine plus 15 mM glutamate for 24 h; SC-siRNA + Ami + Glu: cells incubated with scrambled- (SC-) siRNA transfection complexes for 24 h and then exposed to 500 *μ*M amifostine plus 15 mM glutamate for 24 h. Cell viability and LDH release were evaluated by MTT assay and LDH reagent kit, respectively; cell morphology was observed by a phase contrast microscope. (a) SOD2-siRNA abolished amifostine-induced increase of cell viability. (b) SOD2-siRNA abolished amifostine-induced decrease of LDH release. (c) SOD2-siRNA abolished amifostine-induced amelioration of cell morphology. Results are means ± SD, ^*∗*^ *P* < 0.05, and bar = 20 *μ*m.

MTT, LDH release, and cell morphology were taken to evaluate the injury degree of PC12 cells. We observed that amifostine restored cell viability (Figure 4(a)), reduced LDH release (Figure 4(b)), and maintained cell morphology (Figure 4(c)) in PC12 cells exposed to glutamate, compared with the cells exposed to glutamate only (*P* < 0.05). However, SOD2-siRNA significantly abolished the protections induced by amifostine (*P* < 0.05).

**Figure 5:**
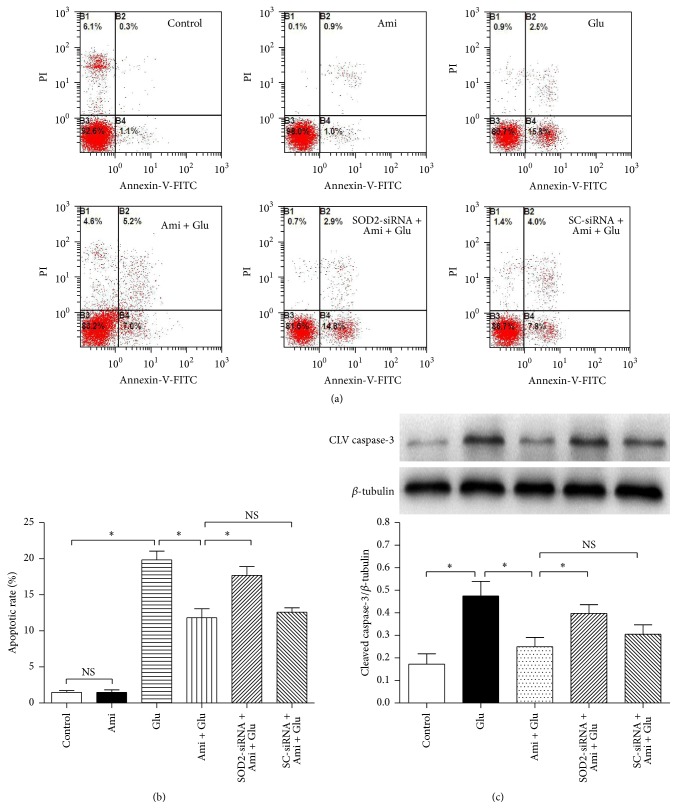
SOD2-siRNA reversed amifostine-induced effects on apoptosis. The PC12 cells were divided into six groups: control: cells cultured in drug-free medium; Ami: cells exposed to 500 *μ*M amifostine for 24 h; Glu: cells treated with 15 mM glutamate for 24 h; Ami + Glu: cells exposed to 500 *μ*M amifostine plus 15 mM glutamate for 24 h; SOD2-siRNA + Ami + Glu: cells incubated with SOD2-siRNA transfection complexes for 24 h and then exposed to 500 *μ*M amifostine plus 15 mM glutamate for 24 h; SC-siRNA + Ami + Glu: cells incubated with scrambled- (SC-) siRNA transfection complexes for 24 h and then exposed to 500 *μ*M amifostine plus 15 mM glutamate for 24 h. Apoptotic rate was determined by a flow cytometry, and the expression of cleaved (CLV) caspase-3 was evaluated by western blot analysis. Results are means ± SD, ^*∗*^ *P* < 0.05, and NS: no significance. (a) Apoptotic rates assessed by flow cytometry. (b) Statistical results of apoptotic rates. (c) SOD2-siRNA abolished amifostine-induced decrease of cleaved caspase-3 expression. Results are means ± SD, ^*∗*^ *P* < 0.05, and NS: no significance.

In addition, to further determine the antiapoptotic ability of amifostine, we assessed apoptotic rate and expression of cleaved caspase-3, an apoptosis-associated protein, by using flow cytometry and western blot analysis, respectively. We found that glutamate exposure increased apoptotic rate (Figures 5(a) and 5(b)) and cleaved caspase-3 expression (Figure 5(c)) in PC12 cells, and coadministration of amifostine markedly reduced the apoptotic rate and cleaved caspase-3 expression. And SOD2-siRNA partially abolished the amifostine-induced effects on apoptotic rate and cleaved caspase-3 expression (*P* < 0.05), indicating the amifostine-induced cytoprotective and antiapoptotic effects against glutamate in PC12 cells may be mediated by SOD2.

### 3.5. SOD2-siRNA Significantly Abolished Amifostine-Induced Effects on Mitochondrial Superoxide, Intracellular ROS, GSH, and CAT

**Figure 6:**
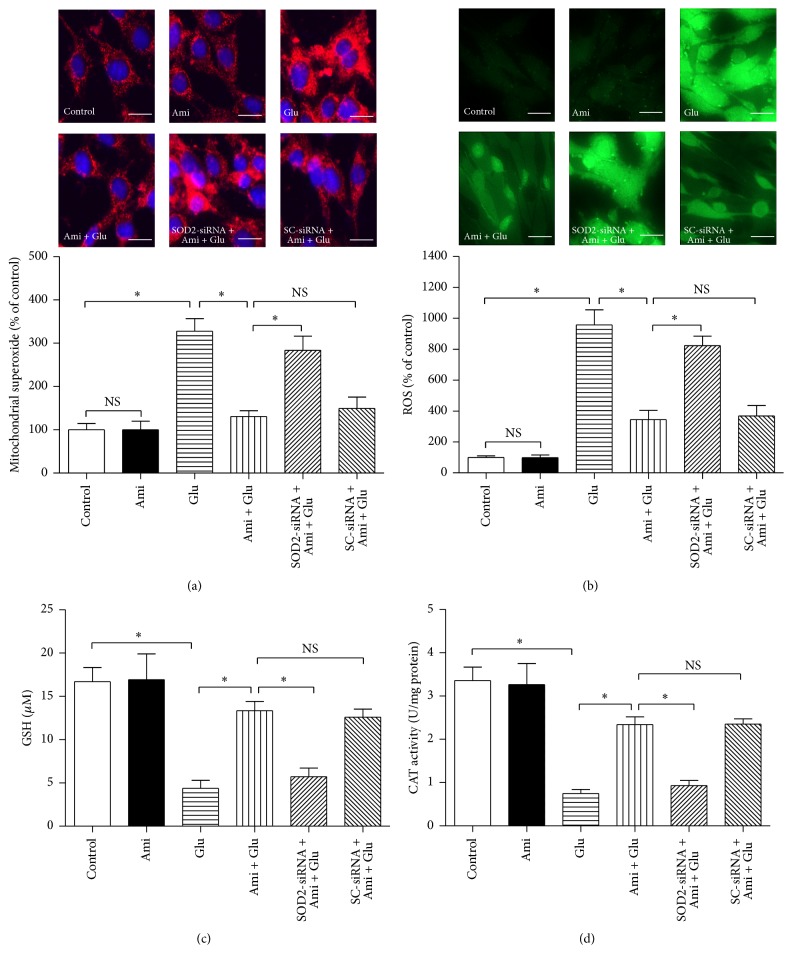
SOD2-siRNA abolished amifostine-induced effects on mitochondrial superoxide production and intracellular ROS, GSH, and CAT levels. The PC12 cells were divided into six groups: control: cells cultured in drug-free medium; Ami: cells exposed to 500 *μ*M amifostine for 24 h; Glu: cells treated with 15 mM glutamate for 24 h; Ami + Glu: cells exposed to 500 *μ*M amifostine plus 15 mM glutamate for 24 h; SOD2-siRNA + Ami + Glu: cells incubated with SOD2-siRNA transfection complexes for 24 h and then exposed to 500 *μ*M amifostine plus 15 mM glutamate for 24 h; SC-siRNA + Ami + Glu: cells incubated with scrambled- (SC-) siRNA transfection complexes for 24 h and then exposed to 500 *μ*M amifostine plus 15 mM glutamate for 24 h. (a) Mitochondrial superoxide production (red) was measured by MitoSOX Red staining followed by image pro-plus software analysis, and nuclei were counterstained with DAPI (blue). Intracellular ROS (b), GSH (c), and CAT (d) levels were evaluated by the corresponding reagent kits, and the photographs of ROS fluorescence staining were recorded by a confocal microscope. Results are means ± SD, ^*∗*^ *P* < 0.05, NS: no significance, and bar = 10 *μ*m.

For detection of mitochondrial superoxide production, we used MitoSOX Red mitochondrial superoxide reagent (Figure 6(a)). Compared with the control, higher mitochondrial superoxide production signals were detected in the cells treated with 15 mM glutamate for 24 h, and amifostine markedly inhibited the glutamate-induced increase of mitochondrial superoxide production signals (*P* < 0.05). However, SOD2-siRNA significantly reversed the amifostine-induced reduction of mitochondrial superoxide production (*P* < 0.05), indicating SOD2 mediates amifostine-induced effects on mitochondrial superoxide production.

**Figure 7:**
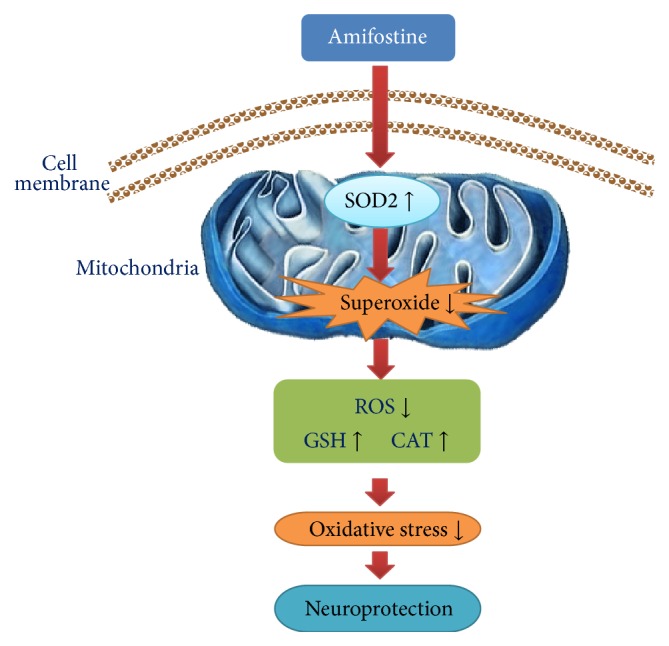
Hypothetical model of SOD2 mediates amifostine-induced neuroprotection against oxidative stress injury. Amifostine exposure increases mitochondrial SOD2 activity, which then consumes mitochondrial superoxide production induced by oxidative stress. Due to the depletion of mitochondrial superoxide production, intracellular ROS level is reduced, and GSH and CAT levels are restored, leading to an inhibition of oxidative stress injury and neuroprotection.

For evaluation of intracellular ROS, GSH, and CAT levels, we used the corresponding reagent kits (Figures 6(b)–6(d)). Administration of amifostine inhibited the increase of intracellular ROS and restored GSH and CAT levels in the PC12 cells exposed to 15 mM glutamate; however, SOD2-siRNA partially abolished the amifostine-induced effects on ROS, GSH, and CAT levels (*P* < 0.05), suggesting SOD2 may mediate amifostine-induced antioxidative actions by maintaining the balance of intracellular oxidants and antioxidants in the glutamate-treated PC12 cells (Figure 7).

## 4. Discussion

In this study, we found that 500 *μ*M amifostine increased SOD2 protein expression, decreased cell injury, inhibited mitochondrial superoxide production and intracellular ROS generation, and restored GSH and CAT levels in PC12 cells exposed to 15 mM glutamate. SOD2-siRNA, however, significantly reversed the amifostine-induced protective and antioxidative effects in the cells, and scramble-siRNA did not cause obvious effects on the amifostine-induced protections. These findings suggest that SOD2 mediates amifostine-induced protections in PC12 cells exposed to glutamate.

Oxidative stress injury is involved in a variety of neurological conditions, including stroke, brain trauma, and neurodegenerative diseases [1–3]; therefore, reducing oxidative injury in CNS is believed to be an effective therapy for the diseases. However, unfortunately, some antioxidants, which are effective in basic studies, did not induce significant antioxidative effects in clinical trials [24–26]. For this reason, searching for safe and effective antioxidants is needed urgently in treating oxidative stress-related diseases. Amifostine is a common used radioprotector drug, approved by the United States Food and Drug Administration (FDA). At present, amifostine is mainly used in reducing the side effects induced by radiation therapy in the patients with malignant tumors [27]. In an* in vivo* study, Merter et al. found that amifostine could attenuate ischemia/reperfusion injury of monkey kidneys by increasing intracellular GSH level [28]. In rat immature hippocampal neurons exposed to *γ*-rays, amifostine protects the neurons against the irradiation injury [29]. However, amifostine has not been used in treating CNS conditions. In this study, we found that amifostine attenuated glutamate neurotoxicity in PC12 cells, indicating amifostine induced neuroprotective effects. In addition, in a mouse model of leg sarcoma, Grdina et al. reported that amifostine protects normal tissue against radiation by increasing intracellular SOD2 level; nevertheless, CAT and glutathione peroxidase (GPx) activities remain unchanged [12]. In another investigation, Murley et al. found that WR1065, the active free thiol form of amifostine, increases SOD2 protein expression in mouse SH-NH sarcoma cells, leading to protection against X-rays-induced oxidative injury. And SOD2-siRNA significantly abolished the WR1065-induced protective effects [13]. Therefore, we investigated the role of SOD2 in amifostine-induced protective effects in this study. We found that SOD2 mediated amifostine-induced antioxidative actions in PC12 cells exposed to glutamate. As SOD2 protein is mainly expressed in mitochondria which have been identified as a major source of ROS [30], we infer that high level of SOD2 protein may protect mitochondria by consuming ROS generated in oxidative injury. Mitochondria, as the “energy factory” of cells, offer adenosine triphosphate (ATP) for the life of cells, and excessive oxidants in mitochondria may cause harm to mitochondrial function and even result in cell death [31, 32]. In this study, we found that amifostine inhibited the increase of mitochondrial superoxide production in glutamate-treated PC12 cells; SOD2-siRNA, however, obviously reversed the amifostine-induced effects on mitochondrial superoxide production, suggesting SOD2 may mediate amifostine-induced effects on mitochondrial superoxide production. In addition, SOD2 mediated amifostine-induced effects on intracellular ROS, CAT, and GSH levels, indicating SOD2 may be the key target of amifostine in maintaining the balance of intracellular oxidants and antioxidants in PC12 cells.

Although amifostine cannot permeate the blood-brain barrier (BBB) [33] in physiological condition, in some pathological conditions, including brain ischemia/reperfusion injury, traumatic brain injury, and neurodegenerative diseases [34–36], the BBB may be destroyed; in this case, the drug may induce protections in neuronal cells. In addition, as some drugs or methods may open the BBB, such as mannitol, borneol, and ultrasound [37–39], combination of amifostine and these drugs or methods may induce significant neuroprotective effects. However, there are still some limitations in our study. First, PC12 cell used in this study is a rat pheochromocytoma cell-line, not neuronal cell; therefore, our findings should be verified in primary cultured neurons and* in vivo*. Second, amifostine is a cytoprotectant, and the drug causes cytotoxic effects at high concentrations; thus, the drug should be used at a moderate dose.

In conclusion, we have demonstrated that amifostine protected PC12 cells against glutamate-induced oxidative injury. Furthermore, we showed that amifostine increased SOD2 protein expression and ameliorated the balance of intracellular antioxidants and oxidants, and SOD2-siRNA significantly reversed the amifostine-induced antioxidative and cytoprotective effects. Our findings indicate that SOD2 mediates amifostine-induced protections in PC12 cells exposed to glutamate.
